# Supplementary material for: EnzML: multi-label prediction of enzyme classes using InterPro signatures
Source: BMC Bioinformatics. 2012 Apr 25;13:61. doi: 10.1186/1471-2105-13-61 (PMC3483700; doi:10.1186/1471-2105-13-61)
Supplement: Addtional file 5 — The Java code to format the data files, evaluate and predict. The file enzml_java_code.tar.gz contains the Java code used to format database data to ARFF and XML formats, to execute cross and train-test (jackknife) evaluations and to record evaluation results to database. More information is included in the readme.txt file and the Javadoc files. The code can be used with a MySQL database. To use a different database software, other JDBC drivers might be required. [file 1471-2105-13-61-S5.gz › java_code/enzml2011/doc/uk/ac/ed/inf/enzml/weka/class-use/AttributeFactory.html]

Uses of Class uk.ac.ed.inf.enzml.weka.AttributeFactory


---


|  |  |  |
| --- | --- | --- |
| **Overview** \| **Package** \| **Class** \| **Use** \| **Tree** \| **Deprecated** \| **Index** \| **Help** | |  |
| PREV   NEXT | **FRAMES**    **NO FRAMES**     **All Classes** |  |


---


## **Uses of Class uk.ac.ed.inf.enzml.weka.AttributeFactory**

| Packages that use AttributeFactory | |
| --- | --- |
| **uk.ac.ed.inf.enzml.mulan** |  |
| **uk.ac.ed.inf.enzml.mulan.attributesfilter** |  |
| **uk.ac.ed.inf.enzml.weka** |  |

Uses of AttributeFactory in uk.ac.ed.inf.enzml.mulan

| Subclasses of AttributeFactory in uk.ac.ed.inf.enzml.mulan | |
| --- | --- |
| `class` | `MulanAttributeFactory`             In the Mulan ARFF, the class attribute is not generated. |

Uses of AttributeFactory in uk.ac.ed.inf.enzml.mulan.attributesfilter

| Subclasses of AttributeFactory in uk.ac.ed.inf.enzml.mulan.attributesfilter | |
| --- | --- |
| `class` | `AttributesFilteredAttributeFactory`             Takes the list of attributes from AttributesFilteredDataSetGenerator, instead of taking it from the instances-attributes map. |

Uses of AttributeFactory in uk.ac.ed.inf.enzml.weka

| Methods in uk.ac.ed.inf.enzml.weka that return AttributeFactory | |
| --- | --- |
| `AttributeFactory` | `DataSetGenerator.getAttributesFactory()` |

---


|  |  |  |
| --- | --- | --- |
| **Overview** \| **Package** \| **Class** \| **Use** \| **Tree** \| **Deprecated** \| **Index** \| **Help** | |  |
| PREV   NEXT | **FRAMES**    **NO FRAMES**     **All Classes** |  |


---
